# The gut microbiota as a potential biomarker for methamphetamine use disorder: evidence from two independent datasets

**DOI:** 10.3389/fcimb.2023.1257073

**Published:** 2023-09-18

**Authors:** Linzi Liu, Zijing Deng, Wen Liu, Ruina Liu, Tao Ma, Yifang Zhou, Enhui Wang, Yanqing Tang

**Affiliations:** ^1^ Department of Psychiatry, The First Hospital of China Medical University, Shenyang, Liaoning, China; ^2^ Department of Psychiatry, The First Affiliated Hospital of Xi’an Jiaotong University, Xi’an, China; ^3^ Department of Psychiatry, Shengjing Hospital of China Medical University, Shenyang, Liaoning, China

**Keywords:** methamphetamine use disorder, gut microbes, machine learning, microbiota-gut-brain axis, addiction

## Abstract

**Background:**

Methamphetamine use disorder (MUD) poses a considerable public health threat, and its identification remains challenging due to the subjective nature of the current diagnostic system that relies on self-reported symptoms. Recent studies have suggested that MUD patients may have gut dysbiosis and that gut microbes may be involved in the pathological process of MUD. We aimed to examine gut dysbiosis among MUD patients and generate a machine-learning model utilizing gut microbiota features to facilitate the identification of MUD patients.

**Method:**

Fecal samples from 78 MUD patients and 50 sex- and age-matched healthy controls (HCs) were analyzed by 16S rDNA sequencing to identify gut microbial characteristics that could help differentiate MUD patients from HCs. Based on these microbial features, we developed a machine learning model to help identify MUD patients. We also used public data to verify the model; these data were downloaded from a published study conducted in Wuhan, China (with 16 MUD patients and 14 HCs). Furthermore, we explored the gut microbial features of MUD patients within the first three months of withdrawal to identify the withdrawal period of MUD patients based on microbial features.

**Results:**

MUD patients exhibited significant gut dysbiosis, including decreased richness and evenness and changes in the abundance of certain microbes, such as *Proteobacteria* and *Firmicutes*. Based on the gut microbiota features of MUD patients, we developed a machine learning model that demonstrated exceptional performance with an AUROC of 0.906 for identifying MUD patients. Additionally, when tested using an external and cross-regional dataset, the model achieved an AUROC of 0.830. Moreover, MUD patients within the first three months of withdrawal exhibited specific gut microbiota features, such as the significant enrichment of *Actinobacteria*. The machine learning model had an AUROC of 0.930 for identifying the withdrawal period of MUD patients.

**Conclusion:**

In conclusion, the gut microbiota is a promising biomarker for identifying MUD and thus represents a potential approach to improving the identification of MUD patients. Future longitudinal studies are needed to validate these findings.

## 1 Introduction

Amphetamine-type stimulants (ATSs) are a highly prevalent group of illicit drugs with an estimated 27 million global users (Unooda, 2022). Methamphetamine is the most commonly used ATS (Unooda, 2022), and methamphetamine use disorder (MUD) has emerged as a considerable global public health concern (Kohno et al., 2020). MUD is a chronic, relapsing brain disease (Leshner, 1997) that influences the physiology of various systems in patients, such as the central neural system and the gastrointestinal system (Prakash et al., 2017). However, current diagnostic criteria for MUD are based on subjective and qualitative symptoms and feelings reported by patients (Fuchs, 2010; Segata et al., 2011). Although urine and blood tests are commonly used to detect the use of methamphetamine (Cruickshank and Dyer, 2009), the results do not necessarily provide a clear indication of MUD (Segata et al., 2011; Prakash et al., 2017). It is possible for individuals who casually use methamphetamine to test positive, despite not exhibiting MUD. Moreover, some MUD patients may not test positive if they have not used the drug during the test window (Oyler et al., 2002). As such, it is critical to explore alternative objective biomarkers and diagnostic models to more accurately assess the presence of MUD, rather than relying solely on measures of recent drug exposure (Segata et al., 2011).

The withdrawal period plays a pivotal role in MUD, as it is critical for determining a patient’s prognosis regarding whether they will achieve sustained recovery or experience a relapse (Zorick et al., 2010). Specifically, the initial three months after withdrawal are of utmost importance, as MUD patients tend to experience worse outcomes, including elevated levels of anxiety, depression, impulsivity, and cravings for methamphetamine, during this period(Wang et al., 2013). In addition, in MUD patients who were undergoing a withdrawal period of within three months, relative glucose metabolism was observed to be higher in the parietal cortex and lower in the striatum and thalamus (Nora D. Volkow et al., 2001). Furthermore, the first 3 months after withdrawal is a crucial period for intervention (McKetin et al., 2012). Moreover, the duration of withdrawal reflects the time of the most recent methamphetamine use by MUD patients, which may not only provide clues for clinical intervention but also for judicial purposes. However, current detection methods are still inadequate for determining the duration of withdrawal and the time of the most recent drug use by MUD patients.

As research on the microbiota-gut-brain axis continues to expand, the role of the gut microbiota in psychiatric diseases has become increasingly evident (Cryan et al., 2019; Qin et al., 2021). Indeed, the gut microbiota has emerged as a promising new biomarker for a range of central nervous system diseases and mental illnesses, including Parkinson’s disease (Nair et al., 2018), schizophrenia (Zhu et al., 2020), and depression (Hu et al., 2019). Recent research has indicated that compared to healthy controls (HCs), MUD patients exhibit significant differences in some gut microbes (Deng et al., 2021; Yang et al., 2021). The gut microbiota may participate in the development of MUD through its release of inflammatory mediators, such as bacterial lipopolysaccharide (Deng et al., 2021; Yu et al., 2023). Moreover, a previous study conducted on rats showed that gut dysbiosis after methamphetamine cessation changed in relation to the duration of withdrawal (Forouzan et al., 2021). Based on the above evidence, we hypothesized that MUD patients exhibit gut dysbiosis, and this dysbiosis may vary depending on the duration of withdrawal. In addition, these gut microbiota dysbiosis features can help identify MUD patients and the amount of time they have been in withdrawal.

The gut microbiota is a multifaceted and ever-changing community of diverse microbes (Cryan et al., 2019). Given its intricacy, machine learning, a valuable technique for analyzing heterogeneous biological data with inherent noise, has been commonly employed in the development of microbiota-based diagnostic models (Namkung, 2020). In this research, our objective was to examine gut dysbiosis among MUD patients and generate a machine-learning model utilizing gut microbiota features to facilitate the identification of MUD patients. Furthermore, in patients with MUD, we attempted to explore the variability in the gut microbiota in MUD patients who underwent varying durations of abstinence and assess the potential of the microbiota as an indicator for identifying the amount of time spent in withdrawal by MUD patients.

## 2 Methods

### 2.1 Participants

Participants with MUD were recruited from the First Compulsory Rehabilitation Center of Shenyang. To ensure the accuracy of MUD diagnosis, MUD participants were required to be 18-60 years of age, meet the Diagnostic and Statistical Manual of Mental Disorders (DSM) 5 criteria for MUD, and have medical records showing at least two positive urine tests with an interval of more than one month. In addition, to clarify the disease and minimize the impact of the drug on the gut microbiota, all MUD participants had a negative urine test for methamphetamine at the time of sampling. HCs were recruited through advertising and had to meet the following inclusion criteria: age of 18-60 years, no history of psychoactive substance use or meeting any DSM-5 diagnosis criteria, and no family history of mental illness. Exclusion criteria were set for all participants to control for factors that could influence the gut microbiota, including infections by specific pathogens, such as HIV, syphilis, hepatitis B or C virus; any use of antibiotics, probiotics, corticosteroids, or immunomodulators within one month prior to sample collection; gastrointestinal diseases such as irritable bowel syndrome; and specific dietary habits such as a preference for high-fat diets or completely vegetable-based diets. This study received approval (No. [2021]361) from the Ethics Committee of the First Hospital of China Medical University and adhered to the principles of the Declaration of Helsinki. All participants provided written informed consent after being fully informed about the study.

### 2.2 Collection and analysis of demographic and clinical information

After signing the informed consent form, participants completed a self-report questionnaire to provide their demographic information, including age, sex, and height. MUD participants also provided information on their history of methamphetamine use, as well as their level of methamphetamine craving using a visual analog scale (VAS). Finally, all participants had their weight measured to determine their body mass index (BMI) accurately. SPSS v24.0 (Corp., 2016) was used to analyze the demographic and clinical information. We used the chi-square test to analyze differences in sex distribution between groups. For continuous variables, we compared differences between groups using statistical analyses, including independent t tests and Mann-Whitney U tests, depending on the distribution properties of the data. As the first three months of withdrawal is a critical period for MUD patients (Guo et al., 2022), they were further grouped according to their withdrawal time. The short-term withdrawal subgroup (withdrawal time < 3 months) and the long-term withdrawal subgroup (withdrawal time >= 3 months) were compared to analyze the gut microbiota features of MUD patients with different withdrawal times.

### 2.3 Sample collection and 16S rDNA sequencing

Fecal samples were collected using fecal DNA storage tubes (CW2654, CwBiotech, Beijing, China) and sent to the laboratory within 72 hours. The samples were stored at -80°C in the laboratory until 16S rDNA sequencing analysis was performed. Sample DNA was extracted using the MN^®^ NucleoSpin 96 Soil Kit following the manufacturer’s instructions. Prior to the polymerase chain reaction, Qubit fluorometric quantitation was used to measure both the quantity and quality of isolated DNA. The bacterial 16S rDNA gene V3-V4 region was amplified by a specific primer pair (338F: 5’-ACTCCTACGGGAGGCAGCA-3’, 806R: 5’-GACTACHVGGGTATCTAATCC-3’). Amplicons were purified by gel electrophoresis, quantified and sequenced on an IIumina HiSeq 2500 sequencing platform using paired-end sequencing with a read length of 2*250 bp. The sequencing data were deposited in the National Center for Biotechnology Information (NCBI) BioProject database under project number PRJNA970410.

### 2.4 Sequence data processing and bioinformatic analysis

**Figure 1:**
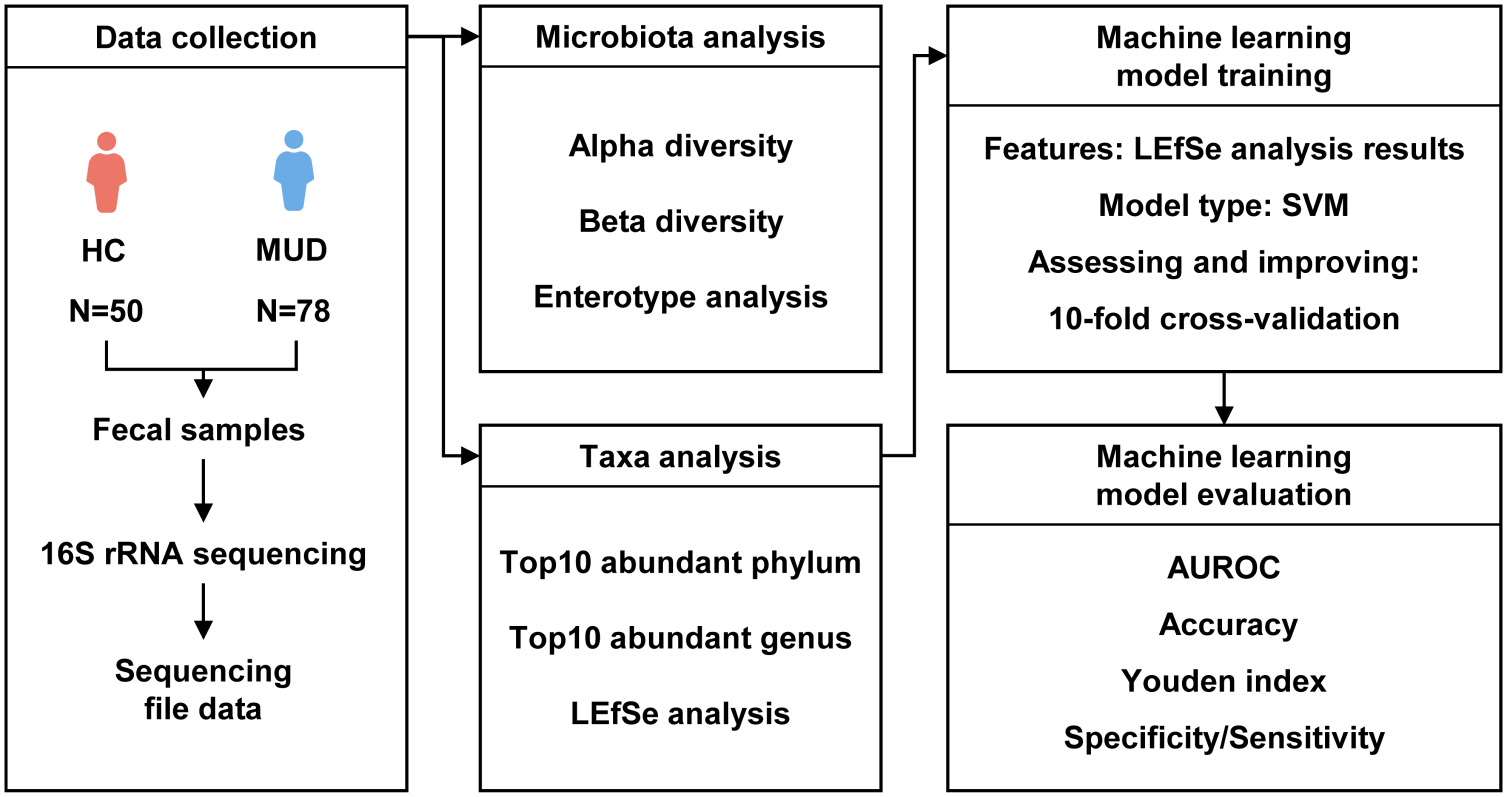
Flow diagram of the development and validation of the MUD patient identification model.

We followed a rigorous pipeline for processing 16S rDNA gene sequencing data (**Figure 1**). FASTP (Chen et al., 2018) was applied to perform adapter and low-quality read filtering using the raw data. Cutadapt v2.7.8 (Martin, 2011) was used to identify and remove primer sequences, resulting in high-quality reads without primer sequences. Then, Trimmomatic v0.33 (Bolger et al., 2014) was utilized to filter the raw reads, resulting in high-quality reads. USEARCH v10.0.240 (Edgar, 2010) and VSEARCH v2.15.2 (Rognes et al., 2016) were used to create amplicon sequence variant (ASV) abundance tables and align sequences against the SILVA database (silva_16S_v123.fa) (Quast et al., 2012) for taxa annotation at different taxonomic levels. Next, we calculated the diversity of the gut microbiota using the vegan package v2.6-4 (Oksanen et al., 2007) in R v4.1.3 (Team, 2022). For alpha diversity analysis, we used the Chao1 index to represent richness and the Shannon index to represent evenness. The Welch t test was used to compare the alpha diversity between the groups. We quantified beta diversity using the Bray-Curtis distance and visualized the results by principal coordinates analysis (PCoA). Permutational multivariate analysis of variance (PERMANOVA) was used to compare the beta diversity between the groups. Enterotypes are a classification system for grouping individuals based on the composition of their gut microbiome. Three main enterotypes, labeled ET_F, ET_B, and ET_P, have been identified (Arumugam et al., 2011). According to the genus-level taxonomic distribution, we analyzed the enterotypes of samples by the enterotype classification model generated by Arumugam, M. et al. (Arumugam et al., 2014) and compared the enterotypes in different groups by the chi-square test. We utilized the Welch t test to compare the abundance of the top 10 phyla and genera between groups with R v4.1.3 and identified potential biomarkers by the linear discriminant analysis (LDA) effect size (LEfSe, LDA score >2.0, p < 0.05) with Galaxy (Segata et al., 2011).

### 2.5 Public data downloading and processing

To create the external dataset, we downloaded 16S rDNA sequencing data from a total of 30 raw fecal samples. These samples were acquired from a published study conducted in Wuhan, China (Yang et al., 2021) (with 16 MUD patients and 14 HCs) from the SRA database (PRJNA679237). After downloading, the taxonomic profiling pipeline was performed according to the process described above.

### 2.6 Machine learning

To develop a machine learning model for MUD diagnosis based on gut microbiota features, we implemented a support vector machine (SVM) using e1071 package v1.7-13 (Dimitriadou et al., 2006) in R v4.1.3 (Team, 2022). Given the superior performance of LEfSe over other feature selection methods, such as stepwise selection, in the field of microbiology (Yang et al., 2020), we chose the relative abundances of the taxa identified as potential biomarkers by LEfSe as our featured variables. The training dataset consisted of samples collected in this study, and the oversampling measurement was used to balance the class distribution. Model tuning was performed using 10-fold cross-validation. To test the generalizability of the classifier, we tested the model in the external dataset obtained from another region.

Moreover, we developed an SVM classifier model for the short-term withdrawal subgroup and long-term withdrawal subgroup. The model features included the relative abundances of taxa identified as potential biomarkers of the short-term and long-term withdrawal groups in LEfSe analysis. The training dataset (90%) was randomly divided from the samples gathered during this study, and oversampling measurements were used to achieve class distribution balance. Model tuning was also carried out via 10-fold cross-validation, and the model was then validated using the remaining 10% of samples in the dataset.

To evaluate the models, we calculated the area under the receiver operating characteristic curve (AUROC), Youden’s index, accuracy, sensitivity, and specificity. The code for the machine learning analysis performed in this study is available at https://osf.io/m5s23/. We considered results to be statistically significant if P < 0.05 in this study.

## 3 Results

### 3.1 Demographic and clinical data

A total of 128 participants were enrolled in the study, including 78 MUD patients and 50 HCs. The average age of the participants was 41.36 ± 9.52 years old, with women comprising 32.8% (n = 42) of the participants. The mean BMI was 25.28 ± 3.24. **Table 1** provides a summary of the characteristics of all study participants. No significant differences were found in age, sex, or BMI between the MUD patients and HCs. Moreover, the median duration of withdrawal among the MUD patients was 48.5 days, while their average course of MUD was 9.24 ± 5.44 years.

**Table 1:** Demographic and clinical information of participants.

Demographic information	HC	MUD	T/χ^2^	P
	n = 50	n = 78		
Female (%)	40	28.2	1.923	0.166
Age (yrs, mean ± SD)	43.40 ± 9.70	40.05 ± 9.23	-1.963	0.052
BMI (kg/m2, mean ± SD)	24.59 ± 3.00	25.71 ± 3.34	1.931	0.056
Withdrawal time (days)	NA	48.5 (19.5, 281.5)	NA	NA
Course of MUD (yrs, mean ± SD)	NA	9.24 ± 5.44	NA	NA
Times of treatment (times)	NA	1 (1, 2)	NA	NA
Craving (VAS scores)	NA	0 (0, 2)	NA	NA

VAS: Visual Analog Scale for craving.

### 3.2 The gut microbiota of MUD patients and HCs

**Figure 2:**
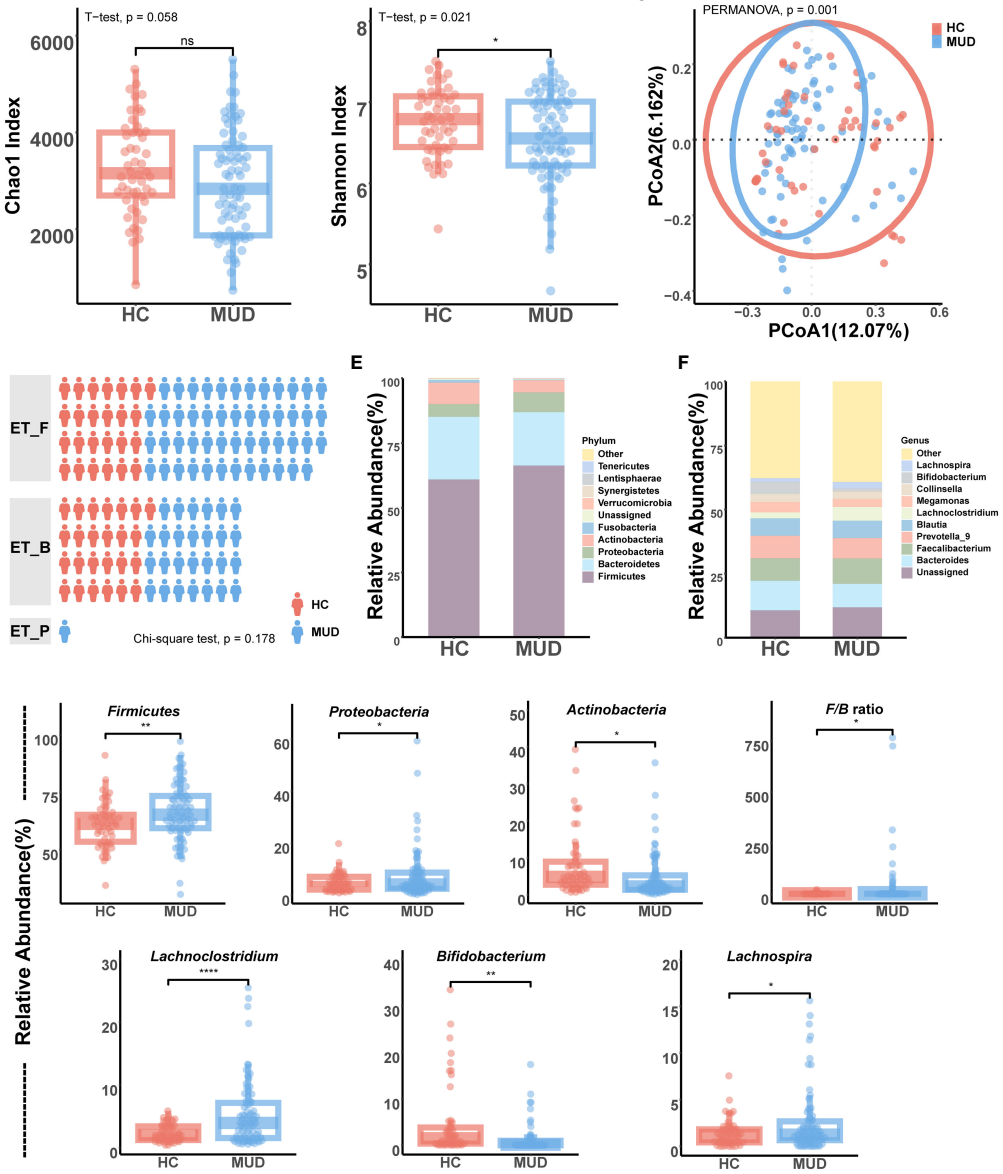
Gut microbiota differences between MUD patients and HCs. **(A)** Comparison of alpha diversity (Chao1) between MUD patients and HCs. **(B)** Comparison of alpha diversity (Shannon) between MUD patients and HCs. **(C)** Comparison of beta diversity (Bray-Curtis distance) between MUD patients and HCs. **(D)** Comparison and distribution of enterotypes in MUD patients and HCs. **(E)** Composition of gut microbiota at the phylum level in the MUD patients and HCs. **(F)** Composition of gut microbiota at the genus level in the MUD patients and HCs. **(G)** Taxa significantly different between MUD patients and HCs in the top 10 abundant phyla/genera. * indicates p < 0.05; ** indicates p < 0.01; **** indicates p < 0.0001; 'ns' indicates p > 0.05.

We read 10,045,147 valid tags from samples collected from the 128 participants. After filtering, denoising, merging, and removing the chimeras and singletons, each sample had an average of 77,709 sequence reads for further analysis. After clustering and aligning with the SILVA database, we identified 22,757 ASVs belonging to 195 genera across 12 phyla. The analysis of alpha diversity showed that MUD patients had a significantly lower Shannon index (T = 2.338, p = 0.021) and a trend of a lower Chao1 index (T = 1.919, p = 0.058) than the HCs (**Figures 2A, B**). Beta diversity performed according to the Bray-Curtis distance also significantly differed (F = 2.254, p = 0.001) between MUD patients and HCs (**Figure 2C**). Upon conducting the enterotype analysis, it was observed that half of the HC group exhibited the ET_F enterotype, while the remaining half possessed the ET_B enterotype. In contrast, MUD patients displayed a higher occurrence rate of the ET_F enterotype, although this finding was statistically nonsignificant, as depicted in **Figure 2D** (χ² = 3.450, p = 0.178).

The taxonomic distribution of gut microbial communities differed between MUD patients and HCs (**Figures 2E, F**). Among the top 10 phyla, the relative abundances of *Firmicutes* (T = -2.700, p = 0.008), *Proteobacteria* (T = -2.247, p = 0.027), and *Actinobacteria* (T = 2.498, p = 0.010) were significantly different between MUD patients and HCs (**Figure 2G**). In addition, MUD patients showed a significantly higher *Firmicutes*/*Bacteroidetes* ratio than HCs (T = -2.384, p = 0.020, **Figure 2G**). Among the top 10 genera, the relative abundances of *Lachnoclostridium* (T = -4.695, p < 0.001), *Bifidobacterium* (T = 3.101, p = 0.003), and *Lachnospira* (T = -2.521, p = 0.010) were significantly different between MUD patients and HCs (**Figure 2G**). Moreover, the MUD patients did not show any significant differences from HCs in the levels of either *Bacteroides* or *Prevotella* (p > 0.05).

### 3.3 Training and testing the MUD identification model

**Figure 3:**
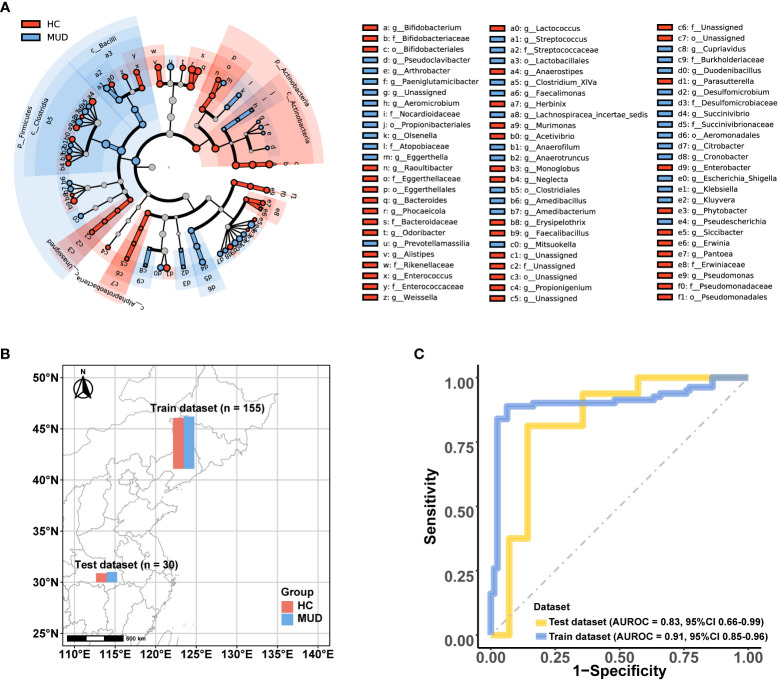
Microbial features and the performance of the MUD patient identification model. **(A)** Cladograms generated by LEfSe (LDA > 2, p < 0.05) indicating differences in the bacterial taxa between MUD patients and HCs, which were used as the microbial features for model training. **(B)** Distribution of the training and test dataset for the MUD patient identification model. **(C)** The AUROC of the MUD identification model using the training and test datasets.

To identify potential biomarkers of MUD, we conducted a LEfSe analysis comparing microbial abundance between MUD patients and HCs (**Figure 3A**; **Supplementary 1**). The results revealed 90 potential biomarkers from five phyla: *Actinobacteria*, *Bacteroidetes*, *Firmicutes*, *Fusobacteria*, and *Proteobacteria*. The abundances of the microbes were used as features in SVM model training. After oversampling, the balanced training dataset included 155 samples (78 MUD patients and 77 HCs, **Figure 3B**). Through 10-fold cross-validation, the model achieved an AUROC of 0.906 (**Figure 3C**), a Youden index of 0.789, an accuracy rate of 0.894, a sensitivity of 0.852, and a specificity of 0.937. Then, we tested the classifier model on the external dataset (samples from Wuhan, China) downloaded from the SRA public database (**Figure 3B**). The classifier showed an AUROC of 0.830 (**Figure 3C**), a Youden index of 0.670, an accuracy rate of 0.833, a sensitivity of 0.813, and a specificity of 0.857.

### 3.4 The gut microbiota of MUD patients with different withdrawal times

**Figure 4:**
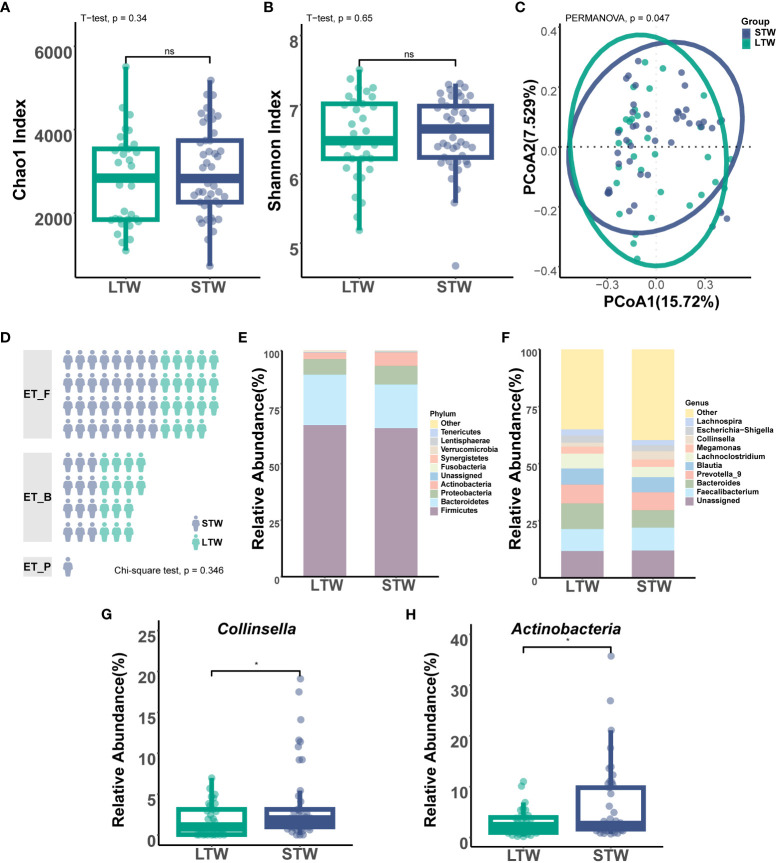
Gut microbiota differences between short- and long-term withdrawal MUD patients. **(A)** Comparison of alpha diversity (Chao1) between short- and long-term-withdrawal MUD patients. **(B)** Comparison of alpha diversity (Shannon) between short- and long-term withdrawal MUD patients. **(C)** Comparison of beta diversity (Bray-Curtis distance) between short- and long-term-withdrawal MUD patients. **(D)** Comparison of the distribution of enterotypes between short- and long-term-withdrawal MUD patients. **(E)** Composition of the gut microbiota at the phylum level in short- and long-term-withdrawal MUD patients. **(F)** Composition of gut microbiota at the genus level in short- and long-term-withdrawal MUD patients. **(H)** Taxa significantly different between short- and long-term withdrawal MUD patients among the top 10 abundant phyla. **(G)** Taxa significantly different between short- and long-term withdrawal MUD patients among the top 10 abundant genera. * indicates p < 0.05; 'ns' indicates p > 0.05. STW indicates short-term withdrawal MUD patients; LTW indicates long-term withdrawal MUD patients.

Based on the withdrawal time, the 78 MUD patients were divided into a short-term withdrawal group (n = 45) and a long-term withdrawal group (n = 33). Other than the withdrawal time, no significant differences were found in demographic and clinical information between the groups (**Supplementary 2**). Microbiota diversity analysis showed that the MUD patients with different withdrawal times were significantly different in beta diversity but not in alpha diversity (**Figures 4A–C**). The enterotype analysis showed that there was no significant difference in the distribution of enterotypes between MUD patients with different withdrawal times (**Figure 4D**, χ² = 2.121, p = 0.346).

**Figure 5:**
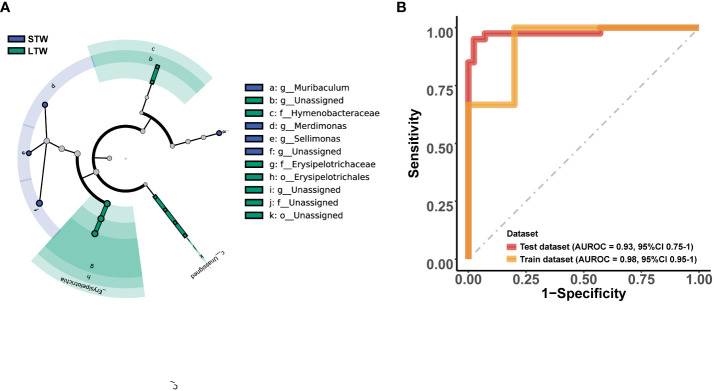
Microbial features and the performance of the MUD withdrawal period identification model. **(A)** Cladograms generated by LEfSe (LDA > 2, p < 0.05) indicating differences in the bacterial taxa between the short-term and long-term withdrawal MUD patients, which were used as the microbial features for model training. **(B)** The AUROC of the MUD withdrawal period identification model on the dataset. STW indicates short-term withdrawal MUD patients; LTW indicates long-term withdrawal MUD patients.

Among the top 10 phyla (**Figure 4E**), only the relative abundance of *Actinobacteria* (T = 2.554, p = 0.013) significantly differed between MUD patients with different withdrawal times (**Figure 4G**). Moreover, there was no significant difference in the *Firmicutes*/*Bacteroidetes* ratio between the two groups (p > 0.05). Among the top 10 genera (**Figure 4F**), only the relative abundance of *Collinsella* (T = 2.386, p = 0.020) significantly differed between groups (**Figure 4H**). LEfSe showed 18 potential biomarkers from four phyla: *Actinobacteria*, *Firmicutes*, *Fusobacteria*, and *Proteobacteria* (**Figure 5A**; **Supplementary 3**). The 18 potential biomarkers were used as features in the withdrawal time classifier training. After random splitting and oversampling, the balanced training dataset included 82 samples (42 short-term withdrawal MUD patients and 40 long-term withdrawal MUD patients). Through 10-fold cross-validation, the model achieved an AUROC of 0.982 (**Figure 5B**), a Youden index of 0.826, an accuracy rate of 0.915, a sensitivity of 0.850, and a specificity of 0.976. Then, we test the model on the test dataset (5 short-term withdrawal MUD patients and 3 long-term withdrawal MUD patients). The classifier showed an AUROC of 0.933 (**Figure 5B**), a Youden index of 0.667, an accuracy rate of 0.875, a sensitivity of 0.667, and a specificity of 1.

## 4 Discussion

Since previous studies have mainly focused on male MUD patients and involved small sample sizes (Deng et al., 2021; Yang et al., 2021), we conducted the largest data-available study to date to compare the gut microbiota composition in 78 MUD male and female patients and 50 sex- and age-matched HCs. We identified 90 taxa from five phyla as potential biomarkers to help identify MUD patients. Based on these taxa, we developed a machine learning model that was highly effective in distinguishing MUD patients. The model also showed generalizability across regions. To further explore the potential role of the gut microbiota in distinguishing MUD patients with different withdrawal stages, we identified 18 taxa as potential biomarkers, and the model based on the taxa also performed well. To our knowledge, this study is the first to provide evidence for the role of the microbiota in MUD identification and to illustrate the differences in the gut microbiota between MUD patients with varying withdrawal times.

In this study, the gut microbiota of MUD patients showed a significant decrease in evenness and a downward trend in richness compared with that of HCs. Additionally, there was a significant difference in beta diversity between the two groups. These findings indicate that individuals with MUD may have an imbalanced gut microbiota. This is consistent with previous studies (Yang et al., 2021; Wang et al., 2023). In addition to diversity, enterotype is an important indicator of the human gut microbiota (Arumugam et al., 2011). This study is the first to compare the enterotype distribution between MUD patients and HCs, and the results showed that MUD patients have a higher percentage of individuals with the ET_F enterotype. Another study found that MUD patients also have a higher percentage of people with the ET_F enterotype than casual methamphetamine users (He et al., 2023). This suggests that the prevalence of the ET_F enterotype may be associated with MUD. One possible explanation is that the gut environment of MUD patients might be more conducive for bacteria associated with the ET_F enterotype to thrive. However, considering the two-way communication between the gut and the brain, there is also a possibility that individuals with the ET_F enterotype have a higher susceptibility to MUD. The taxonomic distribution results revealed additional details regarding the taxa associated with the ET_F enterotype. Among the top 10 genera, *Lachnoclostridium* and *Lachnospira* were significantly enriched in MUD patients, and both were associated with the ET_F enterotype. *Lachnoclostridium* and *Lachnospira* are from the family *Lachnospiraceae*, and their abundance has been found to be positively related to psychotic symptoms in MUD patients, such as difficulty in abstract thinking (Yang et al., 2021). Additionally, it was observed that MUD patients had a significantly higher abundance of *Firmicutes* and a higher *Firmicutes*/*Bacteroidetes* ratio than HCs, which are also features of the ET_F enterotype (Arumugam et al., 2011). An unbalanced *Firmicutes*/*Bacteroidetes* ratio is indicative of gut dysbiosis and is associated with various chronic diseases, such as obesity (Magne et al., 2020), inflammation (Stojanov et al., 2020), and hypertension (Yang et al., 2015). Interestingly, MUD patients are also a high-risk population for these diseases (Kaye et al., 2007; J Edge and Gold, 2011; Davidson et al., 2022). Even though it is challenging to determine a causal relationship, future studies should consider the potential role of the gut microbiota in the increased susceptibility to chronic illnesses among MUD patients. In addition, some taxa, including *Bacteroides* and *Prevotella*, did not differ between the HCs and MUD patients. These two taxa were interpreted as biomarkers of diet and lifestyle (Gorvitovskaia et al., 2016). This suggests that diet and lifestyle factors may have been similar between the HCs and MUD patients in this study. Although our findings demonstrate a significant association between certain gut bacteria and MUD, we have to acknowledge that the study’s design does not allow us to establish causal relationships between changes in the gut microbiota and the development of MUD. To more effectively explore causality, future longitudinal studies would enable us to track changes in both the gut microbiota and MUD development over time, providing more insight into potential causal relationships. Additionally, future interventional trials could involve manipulating the gut microbiota composition and assessing subsequent changes in MUD incidence, offering a more direct way to investigate causality.

In summary, MUD patients showed a notable imbalance in the gut microbiota. To develop a microbiota-based identification model for MUD, we utilized LEfSe to identify potential biomarkers. LEfSe is a commonly utilized method, based on the machine learning model of LDA, to identify biomarkers in the gut microbiome (Segata et al., 2011). Some of the taxa identified by LEfSe have already been shown to potentially contribute to the pathological processes of MUD. For example, *Klebsiella* has been found to influence the lipopolysaccharide level of the host and further mediate the craving for methamphetamine after withdrawal in rats (Yu et al., 2023). *Parasutterella* has been found to be related to the impairment of bile acid homeostasis and host weight gain after methamphetamine use in mice (Zhang et al., 2022). The results related to the gut microbiota features of MUD might provide new perspectives for developing future personalized treatment approaches to reduce drug craving or metabolism imbalance. Based on these potential biomarkers, we developed a machine learning model that performed well (accuracy = 0.894) to help identify MUD patients. There has been no previous study on a microbiota-based model to help identify MUD. What we known about ability of microbiota as the potential biomarker upon previous study is largely based on other diseases. Based on gut microbial biomarkers, Addolorato et al. developed a random forest classifier with an accuracy of 0.934 for identifying patients with alcohol use disorder compared to HCs (Addolorato et al., 2020). Based on oral microbial biomarkers, Kosciolek et al. developed a machine learning model with an accuracy of 0.83 for identifying patients with substance use disorder (including both stimulants and opioids) compared to HCs (Kosciolek et al., 2021). The high-performance metrics of the models were consistent with previous studies on microbial biomarkers for addiction diseases. Considering addiction is a chronic brain disease, another popular potential biomarker is neuroimaging (Yang et al., 2023). In contrast to previous neuroimaging-based models for identifying MUD patients, which had an accuracy range of 0.732-0.880 (Yu et al., 2020; Yan et al., 2021), microbial-feature-based models demonstrate more satisfactory efficacy. Additionally, the generalizability across regions of the model adds another layer of significance. To validate the efficacy and generalizability of our MUD identification model, we introduced an external dataset. Nonetheless, it is crucial to recognize that discrepancies in population demographics, sample collection methods, or laboratory protocols across datasets can collectively influence the model’s performance when extrapolated to an external dataset. For instance, in the external dataset, all samples consisted of male individuals, potentially accounting for the observed decrease in accuracy when the model was applied to this external dataset. However, although the model’s performance when applied to an external dataset declined, it continued to demonstrate promising classification capabilities. The shared effective microbial biomarkers between cohorts from Northwest China (Shenyang) and Central China (Wuhan) in this study suggest that while distinct regional populations might exhibit varying microbial community structures, potential biomarkers for microbial dysbiosis associated with MUD could possess universal attributes. These potential biomarkers manifest a consistent trend in distribution and characteristics of the gut microbiota in MUD patients (Yang et al., 2021; Wang et al., 2023). The results indicate that the microbial changes in MUD patients might be stable across different regions, and the combination of microbiota features and the machine learning model could be an innovative strategy for exploring noninvasive biomarkers for MUD. This approach might help address the issue of subjectivity in current diagnosis techniques based on symptoms (Fuchs, 2010) as well as the limited time window for urine tests (Cruickshank and Dyer, 2009).

While the model’s metrics showcased satisfactory performance, it is necessary to deliberate on the practical significance of its predictive capacity with caution. The inherent limitations of employing machine learning models for clinical outcome prediction cannot be overlooked. First, machine learning models are inherently constrained by the training dataset. Considering that the gut microbiota is notably impacted by ethnicity and environment, even if the model demonstrates strong performance on an external dataset, the applicability of the model to broader contexts is subject to scrutiny. Moreover, machine learning models are frequently regarded as ‘black boxes’ due to the lack of transparency and interpretability. In the realm of clinical practice, it is imperative for the benefit of patients that psychiatrists grasp the underlying rationale for every clinical decision. The models should be regarded as complementary tools, designed to supplement rather than supplant the judgment and expertise of psychiatrists.

To further analyze whether the gut microbiota of MUD patients with different withdrawal times exhibit distinct features, we compared the gut microbiota between long-/short-term-withdrawal MUD patients. Our findings revealed a significant difference in beta diversity and the levels of some taxa, such as *Collinsella*, between long- and short-term withdrawal MUD patients, suggesting that the gut microbiota of MUD patients may change during withdrawal. A previous study conducted on rats also showed that methamphetamine cessation significantly changed the composition of the gut microbiota, and these changes fluctuated depending on the amount of time after cessation (Forouzan et al., 2021). A human research study (Deng et al., 2021) including MUD patients with a longer withdrawal time (an average of 8 months) reported different results in both gut microbiota diversity and composition from studies that included MUD patients with a withdrawal time ranging from 1 week to 6 months (Cook et al., 2019; Yang et al., 2021). Discerning differences among MUD patients who have undergone different withdrawal periods may prove instrumental in identifying the time of the most recent methamphetamine use and recent methamphetamine usage. This is crucial for accurately assessing and further helping provide better MUD management strategies. Our model confirmed that the microbiota differed in MUD patients with different withdrawal periods and indicated that microbiota features may be potential biomarkers to help identify the time of the most recent methamphetamine use by MUD patients, which may further help prompt drug use information in the clinic. However, considering that the sample size of the training dataset was not satisfactory, the model had a risk of overfitting. The result of the MUD withdrawal period identification model should be interpreted with caution.

We acknowledge that there were several limitations to this study. First, the differences in the gut microbiota between long- and short-term-withdrawal MUD patients were based on cross-sectional data, and host factors might have confounded the results. Second, the results of the methamphetamine withdrawal period identification model are exploratory due to the limited sample size for training and lack of an external test dataset. Third, we need to consider the limitations of the study design and specific study populations. Although the results regarding *Bacteroides* and *Prevotella* suggested that diet and lifestyle factors may be similar between HCs and MUD patients, specific information on diet and lifestyle was not collected in this study design. Moreover, the samples included in this study were limited to MUD patients. The results cannot be generalized to other substance use disorders. Fourth, although we analyzed the enterotypes of samples with the enterotype classifier based on the reference dataset of the European Metagenomics of the Human Intestinal Tract project to ensure consistency with previous studies of enterotypes based on large samples, this classification system for gut microbiome enterotypes is still debatable. Additional study on the enterotypes of MUD is needed with the further development of human enterotype research.

In summary, the gut microbiota of MUD patients and HCs differ significantly, and the gut microbiota of MUD patients may change during methamphetamine withdrawal. We developed machine learning models that effectively distinguish MUD patients and determine their withdrawal period, which demonstrated the potential of using gut microbiota as biomarkers in identifying MUD patients. Overall, our results suggest that the fecal microbiome-based model for identifying MUD patients is feasible, which has important implications for the development of noninvasive diagnostics and the establishment of a comprehensive MUD disease management strategy. Future longitudinal studies with larger sample sizes are needed to validate these findings, and multiomics solutions are needed to explore the underlying mechanisms.

## Data availability statement

The data presented in the study are deposited in the Sequence Read Archive, accession number PRJNA970410 (https://www.ncbi.nlm.nih.gov/bioproject/PRJNA970410).

## Ethics statement

The studies involving humans were approved by the Ethics Committee of the First Hospital of China Medical University. The studies were conducted in accordance with the local legislation and institutional requirements. The participants provided their written informed consent to participate in this study.

## Author contributions

LL: Conceptualization, Data curation, Formal Analysis, Investigation, Methodology, Project administration, Visualization, Writing – original draft. ZD: Conceptualization, Data curation, Investigation, Project administration, Writing – review & editing. WL: Investigation, Project administration, Writing – review & editing. RL: Methodology, Supervision, Writing – review & editing. TM: Investigation, Writing – review & editing. YZ: Conceptualization, Supervision, Writing – review & editing. EW: Investigation, Writing – review & editing. YT: Conceptualization, Funding acquisition, Project administration, Resources, Supervision, Writing – review & editing.
