# Supplementary figures and images for: The gut microbiota as a potential biomarker for methamphetamine use disorder: evidence from two independent datasets
Source: Front Cell Infect Microbiol. 2023 Sep 18;13:1257073. doi: 10.3389/fcimb.2023.1257073 (PMC10543748; doi:10.3389/fcimb.2023.1257073)

HC MUD

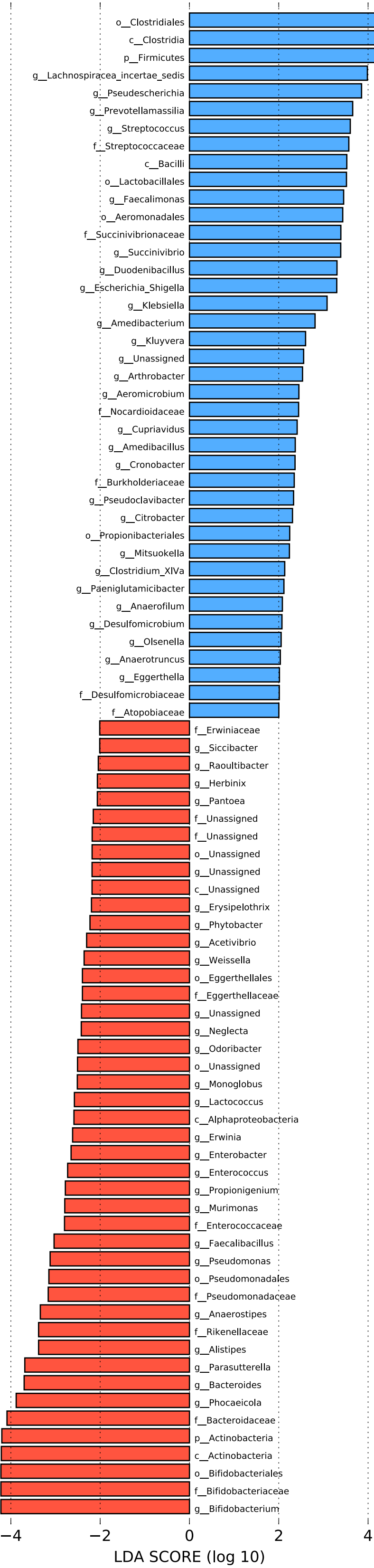

Supplement: Supplementary file 1 [file DataSheet_1.pdf]

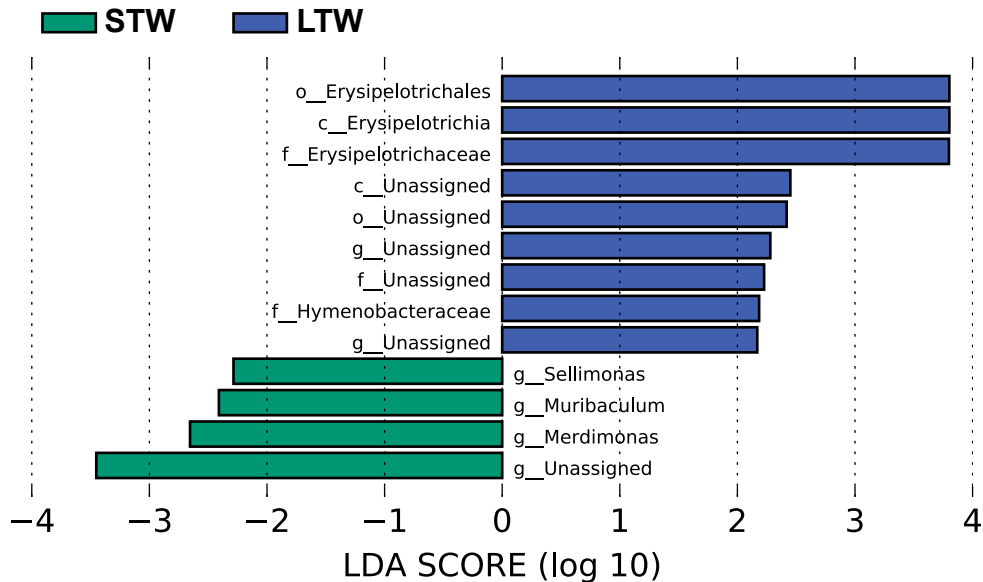

Supplement: Supplementary file 2 [file DataSheet_2.pdf]
