# Supplementary material for: The gut microbiota as a potential biomarker for methamphetamine use disorder: evidence from two independent datasets
Source: Front Cell Infect Microbiol. 2023 Sep 18;13:1257073. doi: 10.3389/fcimb.2023.1257073 (PMC10543748; doi:10.3389/fcimb.2023.1257073)
Supplement: Supplementary file 3 [file Table_1.docx]

Supplementary. 2 Demographics and clinical information of MUD with different withdrawal period.

| Demographic  information | Short-term withdrawal group | long-term withdrawal group | t/t’/χ^2^ | P |
| --- | --- | --- | --- | --- |
|  | n = 45 | n = 33 |  |  |
| Female (%) | 30.3 | 26.7 | 0.124 | 0.724 |
| Age (yrs, mean ± SD) | 40.50 ± 10.00 | 39.42 ± 8.17 | 0.511 | 0.611 |
| BMI (kg/m2, mean ± SD) | 25.98 ± 3.39 | 25.34 ± 3.27 | 0.830 | 0.409 |
| Withdrawal time (days) | 10 (25,36.5) | 317 (173,1077) | -4.970 | <0.001 |
| Course of MUD (years) | 8.17 ± 5.00 | 10.31 ± 5.74 | -1.513 | 0.136 |
| Times of treatment | 1 (1,2) | 1 (1,2) | 0.263 | 0.793 |
| Craving (VAS) | 0 (0,2) | 0 (0,1.5) | 0.482 | 0.632 |

*VAS: Visual Analog Scale for craving.
